# Abscisic Acid Regulates Anthocyanin Biosynthesis and Gene Expression Associated With Cell Wall Modification in Ripening Bilberry (*Vaccinium myrtillus* L.) Fruits

**DOI:** 10.3389/fpls.2018.01259

**Published:** 2018-08-29

**Authors:** Katja Karppinen, Pinja Tegelberg, Hely Häggman, Laura Jaakola

**Affiliations:** ^1^Department of Ecology and Genetics, University of Oulu, Oulu, Finland; ^2^Climate laboratory Holt, Department of Arctic and Marine Biology, UiT The Arctic University of Norway, Tromsø, Norway; ^3^Norwegian Institute of Bioeconomy Research (NIBIO), Ås, Norway

**Keywords:** *Vaccinium*, non-climacteric fruit, berry ripening, hormonal regulation, signaling molecules, anthocyanins, abscisic acid, sucrose

## Abstract

Ripening of non-climacteric bilberry (*Vaccinium myrtillus* L.) fruit is characterized by a high accumulation of health-beneficial anthocyanins. Plant hormone abscisic acid (ABA) and sucrose have been shown to be among the central signaling molecules coordinating non-climacteric fruit ripening and anthocyanin accumulation in some fruits such as strawberry. Our earlier studies have demonstrated an elevation in endogenous ABA level in bilberry fruit at the onset of ripening indicating a role for ABA in the regulation of bilberry fruit ripening. In the present study, we show that the treatment of unripe green bilberry fruits with exogenous ABA significantly promotes anthocyanin biosynthesis and accumulation both in fruits attached and detached to the plant. In addition, ABA biosynthesis inhibitor, fluridone, delayed anthocyanin accumulation in bilberries. Exogenous ABA also induced the expression of several genes involved in cell wall modification in ripening bilberry fruits. Furthermore, silencing of *VmNCED1*, the key gene in ABA biosynthesis, was accompanied by the down-regulation in the expression of key anthocyanin biosynthetic genes. In contrast, the treatment of unripe green bilberry fruits with exogenous sucrose or glucose did not lead to an enhancement in the anthocyanin accumulation neither in fruits attached to plant nor in post-harvest fruits. Moreover, sugars failed to induce the expression of genes associated in anthocyanin biosynthesis or ABA biosynthesis while could elevate expression of some genes associated with cell wall modification in post-harvest bilberry fruits. Our results demonstrate that ABA plays a major role in the regulation of ripening-related processes such as anthocyanin biosynthesis and cell wall modification in bilberry fruit, whereas sugars seem to have minor regulatory roles in the processes. The results indicate that the regulation of bilberry fruit ripening differs from strawberry that is currently considered as a model of non-climacteric fruit ripening. In this study, we also identified transcription factors, which expression was enhanced by ABA, as potential regulators of ABA-mediated bilberry fruit ripening processes.

## Introduction

Fleshy fruits and berries have important roles in human health and nutrition, and therefore their ripening regulation have been intensively studied. Development and subsequent ripening of fleshy fruits are complex processes including major metabolic and structural changes, such as accumulation of pigments, flavor and aroma compounds as well as changes in fruit texture. These processes are controlled by a series of signaling events regulated by plant hormones. Fleshy fruits are physiologically defined as either climacteric or non-climacteric according to the differences in respiration rate and production of plant hormone ethylene during ripening (Osorio et al., 2013). A burst of ethylene accompanied by an increase in the respiration rate at the onset of ripening has long been known to be a critical signal controlling ripening of climacteric fruit, such as tomato, mango, melon, apple, and peach. In contrast, ripening mechanisms of non-climacteric fruits, lacking the burst of respiration and ethylene production, have remained less understood (Cherian et al., 2014).

Studies have shown that plant hormone abscisic acid (ABA), in addition to its central role in plant growth and development and in the adaptation to stress conditions (Vishwakarma et al., 2017), is a major regulator of non-climacteric fruit ripening (Cherian et al., 2014; Leng et al., 2014). ABA has been indicated as a ripening promoter in many non-climacteric fruits, such as strawberry (Jia et al., 2011; Li et al., 2011; Kadomura-Ishikawa et al., 2015), grape (Koyama et al., 2010; Jia et al., 2017), sweet cherry (Luo et al., 2014; Shen et al., 2014), cucumber (Wang et al., 2013), citrus (Zhang et al., 2014), pear (Dai et al., 2014), and litchi (Singh et al., 2014) but also for climacteric fruits, such as tomato, peach, melon, and mango (Zhang et al., 2009; Soto et al., 2013; Sun et al., 2013; Zaharah et al., 2013; Mou et al., 2015). The direct molecular level evidence for the role of ABA in fruit ripening was shown in strawberry by suppressing the expression of the key ABA biosynthetic gene, *FaNCED1*, blocking ABA biosynthesis and leading to partly uncolored strawberry fruits that could be rescued by exogenous ABA (Jia et al., 2011).

Progress has been made during recent years in the understanding of the molecular mechanisms underlying ABA perception and signal transduction during non-climacteric fruit ripening (Li et al., 2011; Cherian et al., 2014; Leng et al., 2014). The ABA receptors were identified by down-regulating the expression of receptors *FaPYR1* and *FaCHLH/ABAR* that led to delay in strawberry fruit ripening and fruit coloring that could not be rescued by exogenous ABA (Chai et al., 2011; Jia et al., 2011). Studies have also identified some ABA-regulated fruit ripening-related transcription factors (TFs) belonging to different gene families, such as MADS, MYB, and bZIP (Daminato et al., 2013; Nicolas et al., 2014; Medina-Puche et al., 2016). However, the ABA-mediated regulatory network promoting non-climacteric fruit ripening still remains poorly understood.

Sugars have traditionally been considered as a carbon and energy source for plants, and in fruits sugars have been thought merely to affect fruit quality. A growing number of studies have indicated that sugars, such as glucose and sucrose, can act as signaling molecules and possess hormone-like signaling functions in plant development and stress responses (Van den Ende and El-Esawe, 2014; Huang et al., 2016). Especially sucrose-specific signaling pathway has been proposed in the regulation of anthocyanin biosynthesis, and in *Arabidopsis* anthocyanin biosynthesis was shown to be up-regulated by sucrose (Solfanelli et al., 2006; Loreti et al., 2008). Fruit ripening signals have been studied extensively in strawberry, which is currently considered as a model of non-climacteric fruit ripening (Li et al., 2011; Cherian et al., 2014). In strawberry, studies have demonstrated that especially sucrose but also glucose promotes fruit ripening (Jia et al., 2011, 2013a,b). Currently, sucrose in co-operation with ABA are indicated as the core signaling molecules regulating strawberry fruit ripening (Jia et al., 2011, 2013b). The coordinated regulation of fruit ripening by ABA and sucrose has recently been suggested also for grapes (Jia et al., 2017).

Bilberry (*Vaccinium myrtillus* L.) is one of the most abundant wild berries in the Northern Europe and it is valued for its nutraceutical and health-beneficial properties (Kolehmainen et al., 2012; Jimenez-Garcia et al., 2013; Törrönen et al., 2013). Ripening of non-climacteric bilberry fruit is characterized by a high accumulation of health-beneficial anthocyanins both in peel and flesh providing deep blue color to the ripe fruits. Anthocyanins are biosynthesized via the well-known phenylpropanoid/flavonoid pathway consisting of a number of enzymatic steps that catalyze a sequential reaction leading to the production of different anthocyanin classes (**Supplementary Figure S1**). Our earlier studies have identified altogether 33 different anthocyanins in bilberry fruits belonging to delphinidin, cyanidin, petunidin, peonidin, and malvidin classes (Zoratti et al., 2014).

So far, there are no studies concerning the role of ABA or sugars on the regulation of fruit ripening and anthocyanin biosynthesis in bilberry fruit. Furthermore, earlier studies have given contradictory results on the role of ABA in the anthocyanin accumulation in other *Vaccinium* species (Percival and MacKenzie, 2007; Forney et al., 2009; Buran et al., 2012; Oh et al., 2018). In our previous study, an increase in endogenous ABA level accompanied by an increase in the expression of *VmNCED1*, the key gene in ABA biosynthesis, was demonstrated at the onset of bilberry fruit ripening suggesting a role for ABA in bilberry ripening regulation (Karppinen et al., 2013).

The aim of the current study was to examine the role of ABA and various sugars on bilberry fruit ripening and ripening-related processes. For this purpose, the effect of exogenous ABA and sugars on bilberry fruit ripening and anthocyanin accumulation was examined in both pre- and post-harvest experiments. The effects of the post-harvest treatments on the expression of the key genes in anthocyanin, ABA and sucrose biosynthesis as well as the expression of the genes encoding major cell wall modifying enzymes was studied. The role of ABA in anthocyanin biosynthesis was further examined by silencing *VmNCED1* in ripening bilberry fruits. Finally, we also identified potential TFs in ABA-regulated bilberry fruit ripening processes.

## Materials and Methods

### Plant Material

Bilberry (*V. myrtillus* L.) plant material used for the experiments was originated from the natural forest stands in Oulu (65°01′ N, 25°28′ E) and Tromsø (69°42′ N, 18°51′ E). For the virus-induced gene silencing (VIGS) experiments, bilberry plants with their root system were harvested at the stage when fruits were unripe and green. The plants were placed in boxes (50 cm × 70 cm) with forest peat soil and watered well. The five developmental stages of bilberry fruit were collected as described earlier (Karppinen et al., 2013).

### Pre-harvest Treatments With ABA and Sugars

In order to study the effect of exogenous ABA on bilberry fruits still attached to plants, an experiment was conducted on field conditions with bilberries growing on the natural forest stand in Oulu, Finland July 2014. For the treatments, ABA [(±)-abscisic acid; Sigma, St. Louis, MO, United States] at concentrations of 0.5 and 2 mM with 0.5% (v/v) Tween 20 were utilized. A solution containing water with 0.5% (v/v) Tween 20 was used as a control treatment. The solutions were applied individually on unripe green bilberry fruits by spraying until run-off with a hand-held sprayer on alternate days for 6 days (three times) in the late afternoon to minimize ABA photo-degradation. Approximately 50 berries were utilized per treatment with four replicates by employing around 15 m^2^ areas adjacent to each other. Berries were evaluated for their color 7 days from the beginning of the first treatment. Berry samples were collected after 0, 24, 48, 96, and 168 h (7 days) from the first treatment, immediately frozen in liquid nitrogen and stored at -80°C until used for RNA extraction and determination of anthocyanin content.

The effect of glucose and sucrose on bilberry fruits still attached to plants was studied on field conditions with bilberries growing on the natural forest stand in Tromsø, Norway August 2015. Sucrose and glucose at concentration of 200 mM with 0.5% (v/v) Tween 20 were used. A solution containing water with 0.5% (v/v) Tween 20 was used as a control treatment. The solutions were applied individually on unripe green bilberry fruits similarly as described above by spraying until run-off with a hand-held sprayer on alternate days for 6 days (three times). Approximately 50 berries per treatment were utilized with four replicate areas as described above. When obvious induction in berry ripening was not detected after 7 days from the first treatment, the treatments were repeated (three times on alternate days). Berries were evaluated for their color and collected after 0, 7, and 19 days from the beginning of the first treatment, immediately frozen in liquid nitrogen and stored at -80°C until used for the determination of anthocyanin content.

### Post-harvest Treatments With ABA and Sugars

For studying the effect of ABA and sugars on detached bilberry fruits, fruits at unripe green stage were harvested from natural forest stand in Oulu, Finland July 2017. Fruits of similar size and color with absence of physical injuries or insect infections were selected for the experiment. The experiment was set-up aseptically under a laminar flow. After rinsing the fruits three times with sterile distilled water, the fruits were randomly divided and immersed with their pedicels (Roubelakis-Angelakis and Kliewer, 1986; Jia et al., 2013a) into the following filter sterilized solutions in sterile Petri plates: 0.5 and 2 mM ABA [(±)-abscisic acid; Sigma], 50 and 200 mM sucrose (VWR International, Lutterworth, United Kingdom), 50 and 200 mM glucose (Sigma), 50 and 200 mM fructose (Merck, United States), 200 μM fluridone (Sigma), 0.5 mM ABA + 200 mM sucrose, and water (control). All solutions contained 0.5% (v/v) Tween 20. Three replicate Petri plates with approximately 50 berries per plate were employed. The plates were placed at 18°C under 30 μmol m^-2^ s^-1^ light. The berries were evaluated for their color on the 4th and 6th day from the beginning of the experiment. Berry samples were collected after 0, 24, 48, 96, and 144 h (6 days) from the beginning of the experiment, immediately frozen in liquid nitrogen and stored at -80°C until used for RNA extraction and determination of anthocyanin content.

### Construction of *VmNCED1* VIGS Vector and *Agrobacterium*-Mediated Infiltration

A 165 bp cDNA fragment of *VmNCED1* (GenBank accession no. JX982599) was PCR-amplified using forward primer 5′-GGATCCCGATCAGCAAGTGGTGTTTA-3′ (*Bam*H1 site is underlined) and reverse primer 5′-TGGAAGCTTAATGTATCCGGACACTCG-3′ (*Hind*III site is underlined) under standard PCR conditions. The PCR product was gel-purified, digested with *Bam*HI and *Hind*III and ligated into pTV00 vector. The resulting pTV00-*VmNCED1* vector was confirmed by sequencing and transformed into *Agrobacterium tumefaciens* strain GV3101 by the freeze-thaw method.

*Agrobacterium*-mediated infiltration by syringe injection with a needle into bilberry fruits was performed as described earlier by Jaakola et al. (2010). Briefly, a 5 ml cultures of *Agrobacterium* strain GV3101 containing pTV00-*VmNCED1* and strain C58c1 containing pBINTRA6 were grown overnight at 28°C in liquid Luria-Bertani (LB) medium (pH 5.6) with appropriate antibiotics. The overnight cultures were inoculated into 50 ml of LB medium containing 10 mM MES, 20 μM acetosyringone and appropriate antibiotics and grown at 28°C until the OD_600_ of the cultures reached 1.0–1.3. The cells were collected by centrifugation (3500 rpm, 5 min, 20°C), resuspended in infiltration buffer (10 mM MgCl_2_, 10 mM MES, 200 μM acetosyringone) to a OD_600_ of approximately 1.5 and incubated at room temperature at least for 2 h. *Agrobacterium* mixture containing pTV00-*VmNCED1* and pBINTRA6 (1:1 ratio) was injected into unripe green bilberry fruits at two spots on the same side of the berry by a 1-ml syringe with a needle. As a control, only *Agrobacterium* with pBINTRA6 was injected into the fruits. The bilberry plants were placed at 18°C with 60% humidity and 125 μmol m^-2^ s^-1^ light intensity. Fruits were evaluated 4 weeks after injection, then frozen in liquid nitrogen and stored at -80°C until used for RNA extraction.

### Isolation of RNA and cDNA Preparation

Total RNA was isolated from berries according to the method described earlier for bilberry (Jaakola et al., 2001). The cDNA was synthesized from the total RNA by using SuperScript III reverse transcriptase (Invitrogen, Carlsbad, CA, United States) according to the manufacturer’s instructions. The cDNA was purified from the contaminating genomic DNA by using the method described by Jaakola et al. (2004).

### Relative Quantification of Gene Expression

Real-time quantitative reverse transcription PCR (qRT-PCR) analyses were performed with a LightCycler 480 instrument and software (Roche Applied Sciences, Indianapolis, IN, United States). The transcript abundance of the genes was detected using a LightCycler^®^ SYBR Green I Master qPCR Kit (Roche). The qRT-PCR conditions were an initial incubation at 95°C for 10 min followed by 40 cycles at 95°C for 10 s, 60°C for 20 s, and 72°C for 10 s. The studied genes we identified from the publicly available *Vaccinium* transcriptome databases. The gene-specific primer sequences used for the qRT-PCR analyses are listed in **Supplementary Table S1**. Glyceraldehyde-3-phosphate dehydrogenase (*VmGAPDH*; GenBank accession no. AY123769) was employed as a reference gene for the relative quantification of the PCR products. The results were calculated with LightCycler^®^ 480 software (Roche), using the calibrator-normalized PCR efficiency-corrected method (Technical note No. LC 13/2001, Roche). The amplification of only one product in qRT-PCR was confirmed by a melting curve analysis.

### Determination of Total Anthocyanins

Frozen berries were ground to fine powder with a mortar and pestle in the presence of liquid nitrogen. Berry powder of 0.1 g was extracted with methanol acidified with 0.1% HCl (v/v) by sonication in the dark for 10 min followed by shaking at room temperature in the dark for 1 h. After centrifugation, the supernatant was collected and the total anthocyanin content was determined according to the pH differential method (Lee et al., 2005, 2008) that has been tested for bilberry material (Dandena et al., 2012). Analyses were performed with three to four biological replicates. The results were expressed as mg (cyanidin-3-glucoside equivalent) g^-1^ fresh weight.

### Statistical Analysis

The quantitative results of gene expression and measurements of anthocyanins in bilberry fruits were analysed either with Student’s *t*-Test or one-way analysis of variance (ANOVA) followed by Tukey’s HSD test by using SPSS Statistics program, version 25 (IBM, New York, NY, United States).

## Results

### Effect of Pre-harvest Treatments With ABA and Sugars on Bilberry Fruit Ripening and Anthocyanin Accumulation

**FIGURE 1:**
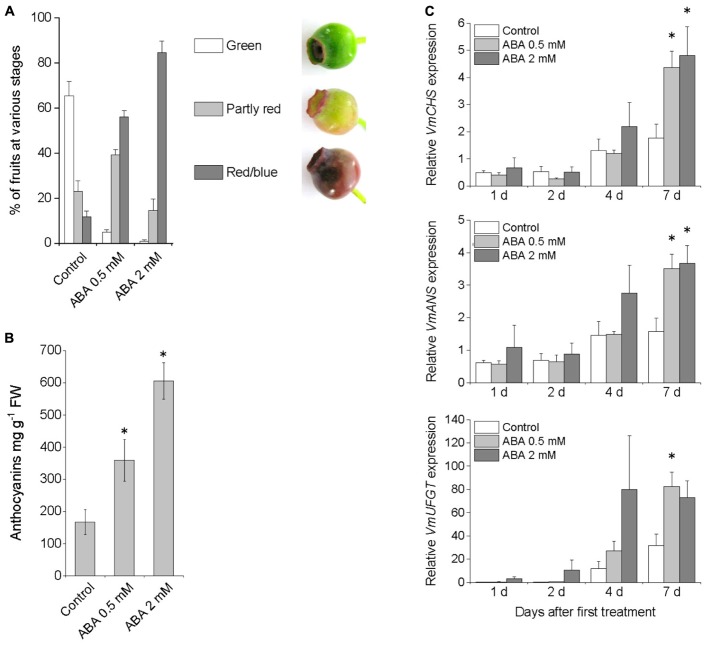
Effect of pre-harvest treatment with ABA on bilberry fruit color **(A)**, anthocyanin content **(B)**, and expression of anthocyanin biosynthetic genes **(C)**. Unripe green berries attached to plants were sprayed with 0.5 mM ABA, 2 mM ABA or water (control). Fruit color and anthocyanin content was evaluated after 7 days from the beginning of the experiment. Total anthocyanin content is expressed as milligrams of cyanidin-3-glucoside equivalents g^-1^ FW. Relative expression of the genes was quantified by qRT-PCR and normalized to *VmGAPDH*. Values represent means ± SEs of four replicates. Asterisks indicate significant differences from control in Student’s *t*-Test (*P* ≤ 0.05).

To investigate the role of ABA on bilberry fruit ripening and anthocyanin accumulation, exogenous ABA was sprayed three times on alternate days on unripe green bilberry fruits still attached to plants. Seven days after the first treatment with 0.5 mM ABA, and especially with 2 mM ABA, most of the fruits had turned red/blue indicating fruit ripening and anthocyanin accumulation while most of the control fruits treated with water were still green (**Figure 1A**). The anthocyanin content was significantly higher at day seven in ABA treated fruits compared to control fruits sprayed with water (**Figure 1B**). Also, both the ABA treatments up-regulated the expression of the anthocyanin biosynthetic genes *VmCHS, VmANS*, and *VmUFGT* during the 7 days experiment (**Figure 1C**).

**FIGURE 2:**
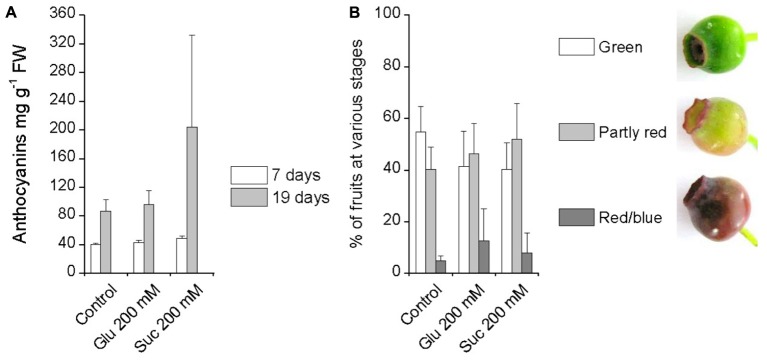
Effect of pre-harvest treatments with glucose and sucrose on bilberry fruit anthocyanin content **(A)** and fruit color **(B)**. Unripe green berries attached to plants were sprayed with 200 mM glucose, 200 mM sucrose or water (control). Total anthocyanin content in fruits was measured after 7 and 19 days from the beginning of the experiment and is expressed as milligrams of cyanidin-3-glucoside equivalents g^-1^ FW. Fruit color was evaluated after 19 days from the beginning of the experiment. Values represent means ± SEs of four replicates.

Glucose and sucrose were similarly applied by spraying on attached unripe green bilberry fruits to investigate their effect on bilberry fruit ripening and anthocyanin accumulation. Seven days after the beginning of the experiment, the fruits were still green in color and neither 200 mM glucose nor 200 mM sucrose had induced significant changes in the fruit anthocyanin content compared to control fruits treated with water (**Figure 2A**). Therefore, the treatments were repeated second time by again spraying berries with sugars three times on alternate days. After 19 days of the first treatment, there were only slightly more red/blue berries in the sugar treatments compared to control berries treated with water (**Figure 2B**). There were no significant differences between the treatments in the anthocyanin content of the berries at day 19, however, there was a high variation between individual berries in the response to sucrose (**Figure 2A**).

### Effect of Post-harvest Treatments With ABA and Sugars on Bilberry Fruit Ripening and Anthocyanin Accumulation

**FIGURE 3:**
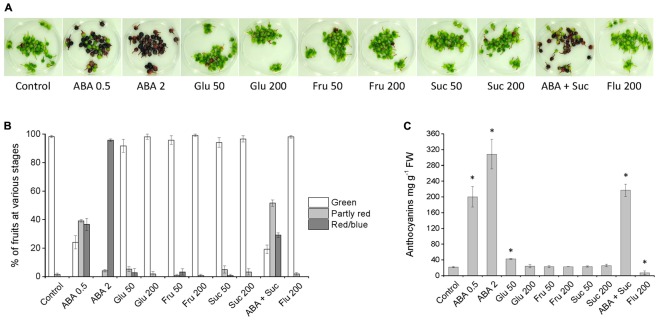
Effect of post-harvest ABA and sugar treatments on bilberry fruit color and anthocyanin accumulation. Detached unripe green berries were immersed into solutions containing ABA (0.5 and 2 mM), glucose (50 and 200 mM), fructose (50 and 200 mM), sucrose (50 and 200 mM), 0.5 mM ABA + 200 mM sucrose, 200 μM fluridone or water (control). After 4 days from the beginning of the experiment, fruits in Petri plates **(A)** were evaluated for their color **(B)** and measured for their anthocyanin content **(C)**. Total anthocyanin content is expressed as milligrams of cyanidin-3-glucoside equivalents g^-1^ FW. Values represent means ± SEs of three replicates. Asterisks indicate significant differences from control in Student’s *t*-Test (*P* ≤ 0.05).

To examine in more detail the role of ABA and sugars on bilberry fruit ripening and anthocyanin accumulation and verify the results attained with attached fruits, unripe green bilberry fruits were harvested and submerged into various post-harvest treatments in Petri plates. The treatments were: ABA (0.5 and 2 mM), glucose (50 and 200 mM), fructose (50 and 200 mM), sucrose (50 and 200 mM), 0.5 mM ABA + 200 mM sucrose, 200 μM fluridone (ABA biosynthesis inhibitor) or water (control). An increase in red coloration in bilberry fruits treated with 2 mM ABA was obvious already after 1 day from the beginning of the treatment. After 4 days, all the fruits in 2 mM ABA treatment and most of the fruits in 0.5 mM ABA and 0.5 mM ABA + 200 mM sucrose treatments had obtained red coloration indicating anthocyanin accumulation (**Figures 3A,B**). The fruits treated with water (control), fluridone or different types and concentrations of sugars were still mostly unripe and green with only few individual berries obtained some red coloration (**Figures 3A,B**). The berries treated with either 0.5 mM or 2 mM ABA had significantly higher levels of anthocyanins compared to control berries in water after 4 days from the beginning of the experiment (**Figure 3C**). The treatment either with glucose, fructose or sucrose, except 50 mM glucose, did not significantly increase the anthocyanin content in bilberry fruits (**Figure 3C**). The increase with 50 mM glucose was due to increase in anthocyanin accumulation in few individual berries and the increase was not seen with 200 mM glucose. Berries treated with fluridone had significantly lower level of anthocyanins compared to control fruits in water (**Figure 3C**). Furthermore, the fruits treated with 0.5 mM ABA + 200 mM sucrose did not have significantly higher anthocyanin level compared to the berries treated only with 0.5 mM ABA, suggesting that sucrose does not significantly enhance the effect of ABA in bilberry anthocyanin accumulation (**Figure 3C**).

After 6 days from the beginning of the experiment, almost all the ABA treated berries had turned fully red/blue while most of the berries in the treatment with water (control), fluridone or sugars were still unripe and green (**Supplementary Figure S2**). None of the berries in fluridone treatment had reached the fully red/blue coloration. The anthocyanin content in berries treated with 0.5 mM ABA had highly increased from day 4. Instead, the anthocyanin accumulation in berries in 2 mM ABA treatment had slowed down from day 4 and the anthocyanin content was significantly lower in berries in 2 mM ABA compared to berries in 0.5 mM ABA treatment (**Supplementary Figure S2**) indicating over-ripening of the berries in 2 mM ABA treatment and cease in anthocyanin biosynthesis.

### Expression of Anthocyanin Biosynthetic Genes in Response to Post-harvest Treatments With ABA and Sugars

**FIGURE 4:**
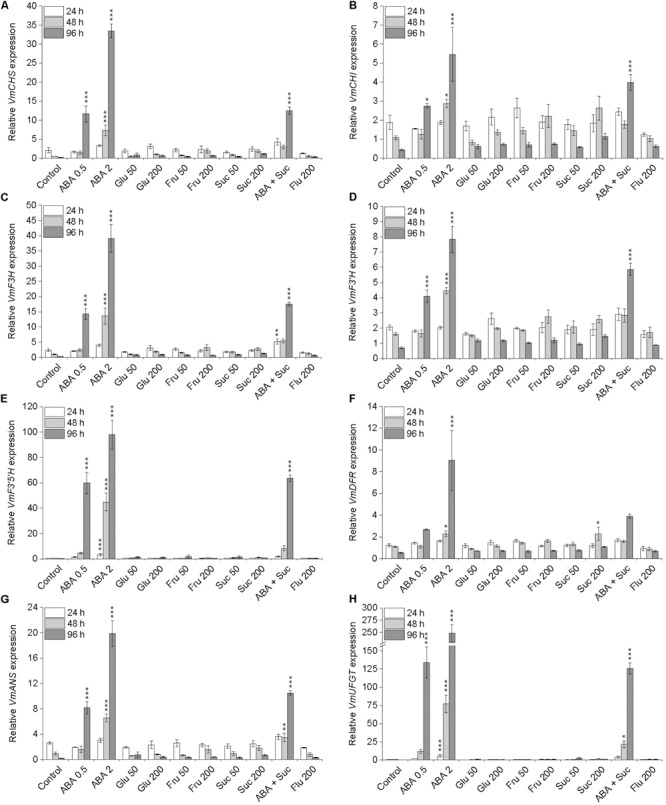
Effect of post-harvest ABA and sugar treatments on the expression of anthocyanin biosynthetic genes *VmCHS* **(A)**, *VmCHI* **(B)**, *VmF3H* **(C)**, *VmF3*′*H* **(D)**, *VmF3*′*5*′*H* **(E)**, *VmDFR* **(F)**, *VmANS* **(G)**, and *VmUFGT* **(H)** in bilberry fruit. The treatments were: ABA (0.5 and 2 mM), glucose (50 and 200 mM), fructose (50 and 200 mM), sucrose (50 and 200 mM), 0.5 mM ABA + 200 mM sucrose, 200 μM fluridone or water (control). Relative expression of the genes was quantified by qRT-PCR and normalized to *VmGAPDH*. Values represent means ± SEs of three replicates. Asterisks indicate significant differences from respective control (^∗^*P* ≤ 0.05, ^∗∗^*P* ≤ 0.01, ^∗∗∗^*P* ≤ 0.001, one-way ANOVA with Tukey’s HSD test).

The transcript levels of anthocyanin biosynthetic genes (**Supplementary Figure S1**) were examined by qRT-PCR in the detached berries during 4 days in different treatments in Petri plates. Significant induction in the expression of all anthocyanin biosynthetic genes was detected in berries in ABA treatments indicating a major role for ABA as a positive regulator of bilberry anthocyanin biosynthesis (**Figure 4**). Especially the transcripts levels of *VmCHS, VmF3H, VmF3′H, VmF3′5′H, VmANS*, and *VmUFGT* were significantly elevated (*P* ≤ 0.001) by both ABA treatments at day 4 and 2 mM ABA treatment already at day 2. For example, after 4 days in 2 mM ABA treatment the up-regulation of *VmCHS, VmF3′5′H*, and *VmUFGT* were 180-, 460-, and 850-fold, respectively, compared to water control. Instead, although slight elevation in gene expression was detected in sugar treatments at day 2 and 4 compared to control fruits, no obvious induction in the expression of the anthocyanin biosynthetic genes in fruits was detected (**Figure 4**). In fact, the expression of the anthocyanin biosynthetic genes was more or less decreased during the 4 days in sugar treatments indicating that sugars have less obvious positive signaling role to induce bilberry anthocyanin biosynthesis compared to ABA (**Figure 4**).

### Expression of ABA and Sucrose Biosynthetic Genes in Response to Post-harvest Treatments With ABA and Sugars

**FIGURE 5:**
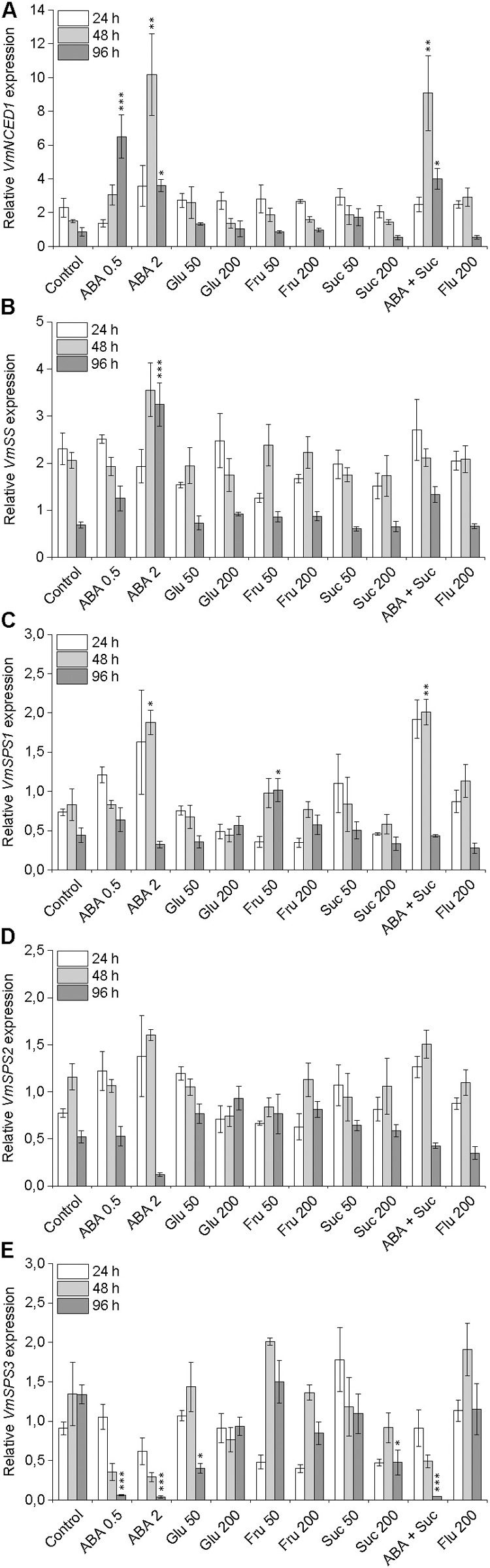
Effect of post-harvest ABA and sugar treatments on the expression of key ABA and sucrose biosynthetic genes *VmNCED1* **(A)**, *VmSS* **(B)**, *VmSPS1* **(C)**, *VmSPS2* **(D)**, and *VmSPS3* **(E)** in bilberry fruit. The treatments were: ABA (0.5 and 2 mM), glucose (50 and 200 mM), fructose (50 and 200 mM), sucrose (50 and 200 mM), 0.5 mM ABA + 200 mM sucrose, 200 μM fluridone or water (control). Relative expression of the genes was quantified by qRT-PCR and normalized to *VmGAPDH*. Values represent means ± SEs of three replicates. Asterisks indicate significant differences from respective control (^∗^*P* ≤ 0.05, ^∗∗^*P* ≤ 0.01, ^∗∗∗^*P* ≤ 0.001, one-way ANOVA with Tukey’s HSD test).

In order to examine the interaction between ABA and sucrose, the transcript levels of ABA and sucrose biosynthetic genes was analyzed in post-harvest bilberry fruits in the different treatments. Bilberry fruits in 0.5 and 2 mM ABA treatments had significantly elevated transcript levels of *VmNCED1*, the key gene in the ABA biosynthetic pathway, compared to control fruits in water (**Figure 5A**) indicating autocatalytic biosynthesis of ABA. In the berries in treatments with different sugars the expression of *VmNCED1* was not induced but decreased during the 4 days experiment. The expression of the two key genes in the sucrose metabolism, *VmSS* and *VmSPS* was also studied, especially in response to ABA treatments. The expression of *VmSS* was significantly elevated in berries in 2 mM ABA treatment compared to control berries after 4 days indicating sucrose degradation while no significant increase in the expression of the gene was observed in other treatments (**Figure 5B**). The expression profiles of the three identified bilberry *SPS* genes slightly differentiated from each other. The expression of *VmSPS1* and *VmSPS2* showed initially up-regulation in berries in ABA treatments and then down-regulation after 96 h compared to control berries, while the *VmSPS3* expression was significantly down-regulated by ABA (**Figures 5C–E**) indicating that ABA does not advance sucrose formation in bilberry fruits.

### Silencing of *VmNCED1* in Bilberry Fruit by Virus-Induced Gene Silencing (VIGS)

**FIGURE 6:**
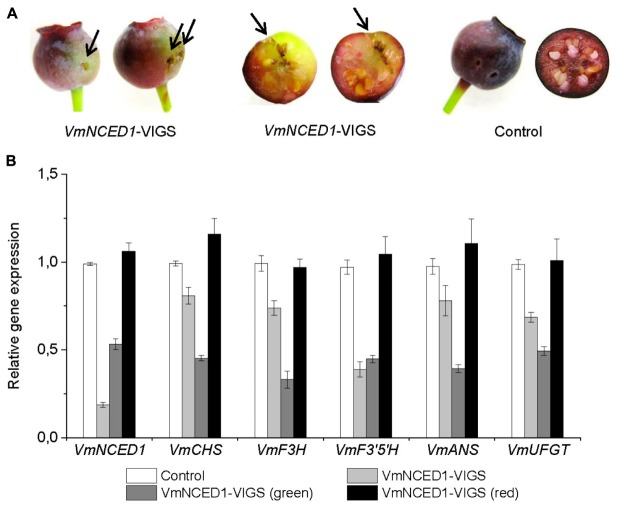
Effect of *VmNCED1* silencing on anthocyanin biosynthesis in ripening bilberry fruit. Green unripe fruits still attached to the bilberry plants were injected with *VmNCED1*-VIGS vector or pBINTRA6 vector only (control). Arrows indicate injection sites. Fruits were evaluated 4 weeks after injection for color **(A)**, and the expression of *VmNCED1* and the key anthocyanin biosynthetic genes in intact fruits as well as in green and red sectors of chimeric fruits **(B)**. Relative expression of the genes was quantified by qRT-PCR and normalized to *VmGAPDH*. Values represent means ± SDs of three replicates.

The effect of ABA on bilberry fruit ripening and anthocyanin biosynthesis was further studied by silencing *VmNCED1*, the key gene in ABA biosynthetic pathway. *VmNCED1*-VIGS vector was injected into unripe green bilberry fruits attached to bilberry plants. After 4 weeks of injection, chimeric fruits with green sectors at the site of injection were found (**Figure 6A**). The transcript levels of the *VmNCED1* were confirmed to be suppressed in these fruits compared to control fruits as well as in green sectors of the chimeric fruits compared to red sectors (**Figure 6B**). The silencing of *VmNCED1* was accompanied by the down-regulation in the expression of anthocyanin biosynthetic genes *VmCHS, VmF3H, VmF3*′*5*′*H, VmANS*, and *VmUFGT* in intact bilberry fruits injected with *VmNCED1*-VIGS vector as well as in green sectors of the fruits compared to red sectors (**Figure 6B**).

### Expression of Genes Associated With Cell Wall Modification in Response to Post-harvest Treatments With ABA and Sugars

**FIGURE 7:**
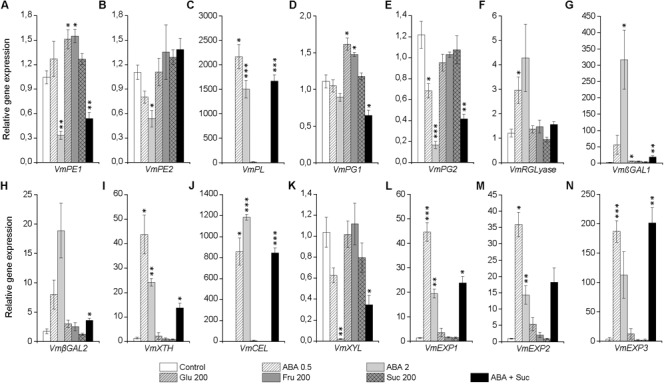
Effect of post-harvest ABA and sugar treatments on the expression cell wall modifying genes *VmPE1* **(A)**, *VmPE2* **(B)**, *VmPL* **(C)**, *VmPG1* **(D)**, *VmPG2* **(E)**, *VmRGLyase* **(F)**, *Vm*β*GAL1* **(G)**, *Vm*β*GAL2* **(H)**, *VmXTH* **(I)**, *VmCEL* **(J)**, *VmXYL* **(K)**, *VmEXP1* **(L)**, *VmEXP2* **(M)**, and *VmEXP3* **(N)** in bilberry fruit. The treatments were: ABA (0.5 and 2 mM), glucose (200), fructose (200 mM), sucrose (200 mM), 0.5 mM ABA + 200 mM sucrose, or water (control). Relative expression of the genes was quantified by qRT-PCR after 4 days of the beginning of the experiment and normalized to *VmGAPDH*. Values represent means ± SEs of three replicates. *PE*, pectin esterase; *PL*, pectate lyase; *PG*, polygalacturonase; *RGLyase*, rhamnogalacturonate lyase; β*GAL*, β-galactosidase; *XTH*, xyloglucan endotransglycosylase/hydrolase; *CEL*, endo-β*-*1,4 glucanase: *XYL*, β-xylosidase; *EXP*, expansin. Asterisks indicate significant differences from control in Student’s *t*-Test (^∗^*P* ≤ 0.05, ^∗∗^*P* ≤ 0.01, ^∗∗∗^*P* ≤ 0.001).

In order study the effect of ABA and sugars on other ripening-related processes, we analyzed the expression of several genes encoding cell wall modifying enzymes in bilberry fruit in response to the post-harvest treatments. As shown in **Figure 7**, ABA significantly increased the expression of some of the genes associated with cell wall modification, including genes indicated in pectin modification *VmPL, VmRGLyase, Vm*β*GAL1*, and *Vm*β*GAL2* as well as genes involved in the depolymerization of hemicellulose *VmXTH, VmCEL*, and three expansins (*VmEXP1, VmEXP2*, and *VmEXP3*). The tested isoforms of *PE, PG*, or *XYL* responded to ABA treatment by down-regulating their expression. Instead, the sugar treatments could elevate expression of some genes associated with cell wall modification and the treatment with glucose and fructose significantly elevated the expression of *VmPE1* and *VmPG1* (**Figure 7**).

### Effect of Post-harvest Treatments With ABA and Sugars on the Expression of Potential Bilberry Fruit Ripening Regulators

**FIGURE 8:**
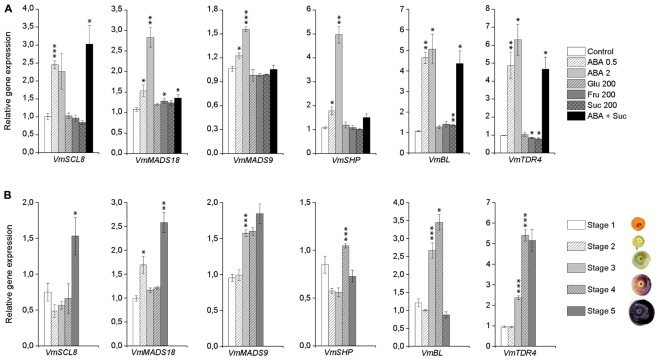
Expression of potential ripening-related transcription factors (TFs) in response to post-harvest ABA and sugar treatments **(A)** and during bilberry fruit development **(B)**. The gene expression was analyzed 4 days after the beginning of the treatments. The treatments were: ABA (0.5 and 2 mM), glucose (200 mM), fructose (200 mM), sucrose (200 mM), 0.5 mM ABA + 200 mM sucrose, or water (control). Relative expression of the genes was quantified by qRT-PCR and normalized to *VmGAPDH*. Values in **(A)** represent means ± SEs of three replicates and asterisks significant differences from control in Student’s *t*-Test (^∗^*P* ≤ 0.05, ^∗∗^*P* ≤ 0.01, ^∗∗∗^*P* ≤ 0.001). Values in **(B)** represent means ± SEs of four replicates and asterisks significant increase from previous developmental stage in Student’s *t*-Test (^∗^*P* ≤ 0.05, ^∗∗^*P* ≤ 0.01, ^∗∗∗^*P* ≤ 0.001). Stages 1–5 indicate the bilberry fruit developmental stages from flower to ripe berry.

In order to get an insight into the ABA signaling transduction in bilberry fruit ripening-related processes, we identified from the publicly available *Vaccinium* transcriptome databases the closest homologs for the genes encoding TFs that have earlier been demonstrated to have a role in ripening regulation or anthocyanin biosynthesis in other fruits. From the tested TFs, expression of *VmSCL8, VmMADS18, VmMADS9, VmSHP*, and *VmBL* were significantly up-regulated by ABA indicating their potential involvement in ABA-regulated fruit ripening processes in bilberry (**Figure 8A**). Also *VmTDR4*, that has earlier been shown to be involved in anthocyanin accumulation in ripening bilberry fruit (Jaakola et al., 2010), was significantly up-regulated by ABA treatments. Expression of *VmMADS18* and *VmBL* were also significantly up-regulated by fructose and sucrose, respectively (**Figure 8A**).

The expression of these TFs was further analyzed during bilberry fruit development and ripening. The expression of *VmSCL8* and *VmMADS18* was significantly elevated in ripe fruit (*VmMADS18* also at stage 2) indicating a role in ripening processes at late stage of bilberry fruit ripening (**Figure 8B**). The expression of the genes *VmMADS9, VmSHP*, and *VmBL* was significantly up-regulated (*P* ≤ 0.001) earlier during the fruit development and at the onset of fruit ripening together with *VmTDR4* (**Figure 8B**).

## Discussion

### ABA Is a Positive Regulator of Bilberry Fruit Ripening Processes

A central role for the plant hormone ABA in promoting fruit ripening has been demonstrated during recent years. Exogenous application of ABA has been shown in many studies to advance especially non-climacteric fruit ripening and the associated anthocyanin accumulation in grape berries (Wheeler et al., 2009; Koyama et al., 2010; Villalobos-González et al., 2016), strawberries (Chai et al., 2011; Jia et al., 2011; Kadomura-Ishikawa et al., 2015; Chen et al., 2016), sweet cherries (Luo et al., 2014; Shen et al., 2014), and litchi fruit (Wei et al., 2011; Singh et al., 2014). Moreover, treatments with inhibitors of ABA biosynthesis, such as fluridone and nordihydroguaiaretic acid (NDGA), delay fruit ripening and decrease anthocyanin accumulation (Jia et al., 2011; Shen et al., 2014; Kadomura-Ishikawa et al., 2015).

In bilberry, the accumulation of anthocyanin pigments is an important indicator of fruit ripening. Our previous studies have demonstrated an increase in ABA content and ABA biosynthesis at the onset of bilberry fruit ripening preceding anthocyanin accumulation (Karppinen et al., 2013, 2016). Similarly, Zifkin et al. (2012) demonstrated a substantial increase in ABA level in highbush blueberries (*V. corymbosum*) at the initiation of fruit ripening suggesting a role for ABA in fruit ripening regulation. However, contradictory results on the effect of ABA in anthocyanin accumulation have been reported when fruits of genus *Vaccinium* have been treated with ABA. Oh et al. (2018) demonstrated that exogenous application of ABA increased northern highbush blueberry (*V. corymbosum*) fruit coloration and accumulation of anthocyanins, especially malvidin, delphinidin, and petunidin glycosides. Instead, exogenous ABA application delayed ripening of southern highbush blueberries (*V. darrowii*; Buran et al., 2012) while ABA had no effect on the anthocyanin accumulation in lowbush blueberry (*V. angustifolium*; Percival and MacKenzie, 2007) or in white cranberries (*V. macrocarpon*; Forney et al., 2009).

The data from the current study clearly demonstrates that ABA induces bilberry fruit anthocyanin biosynthesis. Exogenous ABA applied to unripe bilberry fruits either as pre- or post-harvest treatment promoted berry coloration and anthocyanin accumulation. In addition, the post-harvest treatment with ABA biosynthesis inhibitor, fluridone, delayed bilberry fruit coloration and reduced fruit anthocyanin content. The anthocyanin accumulation in bilberry fruit was supported by the gene expression data demonstrating that all the genes related to anthocyanin biosynthesis were significantly induced by ABA. Especially the expression of *VmUFGT* and *VmF3*′*5*′*H* were highly induced in bilberry fruit by post-harvest ABA treatment. At the branch point in flavonoid biosynthetic route, F3′H leads to cyanidin-derived anthocyanins while F3′5′H activity leads to delphinidin-derived compounds (**Supplementary Figure S1**). Earlier, exogenous ABA treatment has been shown to modify fruit anthocyanin profile (Koyama et al., 2010; Singh et al., 2014; Ju et al., 2016). Our results may imply that in bilberry ABA promotes especially biosynthesis of delphinidin-derived compounds similarly as reported in highbush blueberries by Oh et al. (2018). Also in grape berries, the treatment with high ABA concentration increased the ratio of delphinidin-derived anthocyanins to cyanidin-derived anthocyanins (Ju et al., 2016). The earlier reported contradictory results concerning the role of ABA as a regulator of anthocyanin accumulation among different *Vaccinium* fruits may reflect the differences among the species or a dose of ABA. Also, timing of ABA application has been reported to be critical in ripening promotion among other fruits (Soto et al., 2013; Wang et al., 2013; Luo et al., 2014).

Our results of silencing of *VmNCED1* gene also evidenced the role of ABA in bilberry anthocyanin biosynthesis. 9-*cis*-epoxycarotenoid dioxygenase (NCED), catalyzing the oxidative cleavage of 9-*cis*-isomers of violaxanthin and neoxanthin to xanthoxin, is considered as the key enzyme responsible for ABA biosynthesis (Leng et al., 2014), also in fruits of genus *Vaccinium* (Zifkin et al., 2012; Karppinen et al., 2013). In the present study, silencing of *VmNCED1* in bilberry fruits by virus-induced gene silencing (VIGS) resulted in chimeric fruits with green sectors at the site of infection. The silencing of *VmNCED1* was accompanied by the down-regulation in the expression of the key anthocyanin biosynthesis genes. Our results are similar to previously reported. Previously, Shen et al. (2014) showed that silencing of *PacNCED1* led to the decrease in anthocyanin biosynthesis and resulted in partly colorless sweet cherries. In strawberries, the down-regulation of *FaNCED1* by VIGS was demonstrated to reduce ABA accumulation, delay fruit ripening and anthocyanin biosynthesis, and to lead partly uncolored fruits (Jia et al., 2011; Medina-Puche et al., 2014; Kadomura-Ishikawa et al., 2015). Furthermore, the suppression of *SlNCED1* expression in tomato has been shown to slow down ripening, elongate fruit shelf life and enhance fruit firmness (Sun et al., 2012; Ji et al., 2014).

In the current study, post-harvest ABA treatment was also found to increase expression of *VmNCED1* indicating that ABA regulates its own biosynthesis in bilberry fruit. Earlier, similar results have been reported and externally applied ABA has been found to elevate *NCED* gene expression and ABA synthesis in grapes (Wheeler et al., 2009), cucumber (Wang et al., 2013), and sweet cherry (Luo et al., 2014). Up-regulation of *FaNCED1* expression by ABA has also been demonstrated in strawberry fruits (Chen et al., 2016; Medina-Puche et al., 2016) and it was suggested that the autocatalytic biosynthesis of ABA may be necessary for the induction of high increase in ABA production at fruit ripening (Medina-Puche et al., 2016).

Both transcriptomic and proteomic level studies have revealed that ABA regulates many aspects of non-climacteric fruit ripening (Giribaldi et al., 2010; Li et al., 2015; Medina-Puche et al., 2016; Rattanakon et al., 2016). In addition to pigment formation, fruit ripening is associated with other ripening-related processes, including fruit softening. Structural changes in cell wall polysaccharides pectin, hemicellulose and cellulose due to the action of hydrolytic enzymes and expansins lead to a loss of firmness of fruit pulp at late stage of fruit ripening (Goulao and Oliveira, 2008; Payasi et al., 2009). Studies in non-climacteric grapes (Koyama et al., 2010), strawberry (Li et al., 2014), and cherries (Luo et al., 2014) have implicated that the fruit ripening-related softening is regulated by ABA, and in northern highbush blueberry exogenous ABA was shown to decrease fruit firmness (Oh et al., 2018). In climacteric tomato, involvement of ABA in cell wall degradation was proven by silencing of *SlNCED1* leading to higher pectin content, enhancement in fruit firmness and down-regulation in expression of genes encoding cell wall degrading enzymes (Sun et al., 2012).

Bilberry fruit has a short post-harvest shelf life due to relatively rapid softening. However, gene expression associated with ripening-related fruit softening has not been studied previously in bilberry or other *Vaccinium* species. In the present study, post-harvest ABA treatment during bilberry fruit ripening led to the induction in the expression of genes associated with cell wall modifications. Among these, ABA induced genes encoding pectin-modifying enzymes *VmPL, VmRGLyase, Vm*β*GAL1*, and *Vm*β*GAL2* as well as genes involved in depolymerization of hemicellulose, including *VmXTH, VmCEL*, and three expansins (*VmEXP1, VmEXP2*, and *VmEXP3*). Earlier, fruit softening related gene expression has been studied extensively in strawberry having also a short shelf life. Indications that ABA activates expression of *FaPL, FaCEL, FaRGlyase, FcXTH1, Fa*β*Gal4, FaXYL1* and expansins have been reported (Bustamante et al., 2009; Molina-Hidalgo et al., 2013; Opazo et al., 2013; Chen et al., 2016; Medina-Puche et al., 2016; Paniagua et al., 2016). However, earlier studies have shown that the textural changes occurring during fruit ripening are characteristic to particular fruit species, and are due to differences in type and extent of cell wall modifications and the expression of the modifying enzymes during ripening (Goulao and Oliveira, 2008; Payasi et al., 2009). Overall, our results indicate that the expression of many genes involved in cell wall disassembly is enhanced during bilberry fruit ripening by ABA while the expression of some of the genes/gene isoforms is not induced by ABA.

### Sugars Do Not Induce Bilberry Fruit Ripening Processes

Soluble sugars have been indicated during recent years as fruit ripening regulators in some non-climacteric fruits acting as signaling molecules rather than a carbon source. The role of sugars in fruit ripening has been studied in strawberry and grapes where especially sucrose has been shown to accelerate fruit ripening and anthocyanin biosynthesis. In strawberry, the signaling function of sucrose in fruit ripening was proposed when exogenous sucrose injected at 50 mM dramatically accelerated the fruit ripening while glucose had smaller but also obvious role in ripening and anthocyanin accumulation (Jia et al., 2011, 2013b). Exogenous sucrose at 100 mM was shown to accelerate strawberry fruit ripening in both pre- and post-harvest experiments with injected and immersed berries, respectively (Jia et al., 2013a). Similar results have been reported for some grape varieties (Lecourieux et al., 2014). Recently, Jia et al. (2017) showed that treatments with sugars, especially sucrose, induced anthocyanin biosynthesis and fruit softening in detached Fujiminori grapes. Also, Zheng et al. (2009) demonstrated elevated anthocyanin biosynthesis and accumulation in Cabernet Sauvignon grape berry disks after immersion in solutions containing 50–200 mM glucose, sucrose, or fructose. Spraying with sucrose either alone or in combination with ABA has been shown to increase anthocyanin biosynthesis and accumulation in Crimson Seedless grape berries (Ferrara et al., 2015; Olivares et al., 2017).

The role of sugars in bilberry fruit ripening and anthocyanin biosynthesis has not been studied earlier. Based on the results of the present study, immersion of bilberry fruits into 50 mM or 200 mM glucose, fructose, or sucrose solution does not induce anthocyanin biosynthesis and accumulation but slightly elevates the expression of some genes associated with cell wall modification. Furthermore, sucrose in the ABA + Suc treatment did not enhance the ripening response induced by ABA alone. These results indicate that the role of sugars in the regulation of bilberry fruit ripening differentiates from that reported to strawberry and grapes, and sugars seem to have less important regulatory role in bilberry fruit ripening. However, intensity of the response may also vary between different studies based on the application method of the sugar solutions.

A cross-signaling between ABA and sucrose in anthocyanin biosynthesis has been suggested (Loreti et al., 2008). In strawberry fruit, exogenous sucrose has been shown to stimulate ABA accumulation by promoting dramatically the expression of *FaNCED* (Jia et al., 2011, 2013a,b). Also, silencing of sucrose transporter *FaSUT1* in strawberry led to a decrease in both sucrose and ABA content indicating connection between the two signaling routes (Jia et al., 2013b). In fact, a model for interaction of these two signaling molecules as the core mechanism in regulation of strawberry fruit ripening was recently suggested (Cherian et al., 2014; Jia et al., 2016). Jia et al. (2013b) proposed that sucrose functions as a signal upstream of ABA and induces strawberry fruit ripening both through ABA-dependent and ABA-independent pathways. The effect of ABA on sugar metabolism has also been shown in grape fruits (Çakir et al., 2003; Pan et al., 2005).

However, in the current study, sucrose or other sugar treatments had no inducing effect on *VmNCED1* expression suggesting that sugars do not induce ABA biosynthesis in bilberry during fruit ripening. We also studied the effect of ABA on sucrose biosynthetic gene expression. These results do not support the assumption that ABA might induce sucrose biosynthesis in bilberry fruit. Sucrose phosphate synthase (SPS), catalyzing sucrose synthesis, and sucrose synthase (SS), catalyzing reversible conversion of sucrose to monosaccharides, are indicated as the key enzymes affecting sucrose accumulation in different fruits (Choudhury et al., 2009; Dai et al., 2011; Feng et al., 2012; Li et al., 2012; Tian et al., 2012; Jia et al., 2016). High rate of sucrose accumulation during strawberry ripening was demonstrated to be accompanied by the high expression level of *FaSPS* genes and low expression of *FaSS* gene (Jia et al., 2016). Furthermore, *FaSS1* was suggested as important regulator of strawberry fruit ripening which expression was significantly inhibited by ABA and sucrose treatments (Zhao et al., 2017). Based on our studies, ABA does not induce expression of the *VmSPS* genes in bilberry fruit but increases expression of *VmSS* that is contrast to the results reported in strawberry (Zhao et al., 2017).

Overall, our results indicate that the role of sugars in bilberry fruit differs from strawberry, the current model of non-climacteric fruit ripening. In bilberry fruit, glucose, fructose, or sucrose seem not to act as major signaling molecules to clearly regulate and induce anthocyanin biosynthesis. Despite that strawberry is not an ovary-derived fruit and, thus, considered as a false fruit deviating from bilberry fruit, the regulatory mechanisms of fruit development and ripening has been considered to be conserved among angiosperms (Daminato et al., 2013; Karlova et al., 2014). In order to further clarify the fruit ripening regulation and signaling in bilberry, the signaling route of ABA-mediated bilberry fruit ripening needs to be studied in more detail in the future.

### ABA Up-Regulates Expression of Potential Bilberry Fruit Ripening Regulators

Several types of TFs belonging to different families have earlier been identified as regulators of fruit ripening and anthocyanin biosynthesis, and some of them are regulated by ABA. Fruit ripening regulation has been studied extensively in tomato and in fruits of Rosaceae family. In the current study, we aimed to identify potential TFs of ABA-regulated bilberry fruit ripening processes by searching the closest homologs of functionally characterized TFs of fruit ripening/anthocyanin biosynthesis from publicly available *Vaccinium* transcriptome libraries. Some of the TFs showed highly increased expression in bilberry fruits after ABA treatment. Furthermore, we found elevated TF transcript levels in ripening or ripe fruits indicating a potential role in bilberry fruit ripening-related processes.

One of them was *VmSCL8*, the closest bilberry homolog for *FaSCL8* that is similar to *AtSCL8* in *Arabidopsis*, a member of SCARECROW-LIKE gene family, which members are known to have general roles in plant development. *FaSCL8* expression has been shown to be induced in strawberry receptacle at fruit ripening increasing further in ripe red fruit indicating a role as a regulator of fruit ripening (Pillet et al., 2015). Furthermore, silencing *FaSCL8* in strawberry resulted in lower transcript accumulation of *PAL, CHS, CHI, F3H, UFGT*, and *MYB10* but increased *F3*′*H* and *ANR* transcripts suggesting a role as a general modulator of flavonoid pathway possibly affecting cyanidin-pelargonidin balance by enhancing expression of flavonoid regulating MYB TFs (Pillet et al., 2015). Recently, Medina-Puche et al. (2016) showed that the *FaSCL8* expression in strawberry is elevated by ABA. In the current study, *VmSCL8* also showed elevated expression after ABA treatment. The expression of *VmSCL8* was highest in ripe bilberry fruit similarly to *FaSCL8* indicating a possible role in the ABA-regulated bilberry fruit ripening at the late stages of ripening.

Similarly, elevated expression after ABA treatments in bilberry fruits was observed for three MADS-box genes, *VmMADS18, VmMADS9*, and *VmSHP*, the bilberry homologs for *PyMADS18, FaMADS9*, and *FaSHP*, respectively. MADS-box genes represent highly conserved TF family in plants and have been shown to play important roles in floral and fruit development. While the expression of *VmMADS18* was high in this study both in small green and ripe bilberry fruit, the expression of *VmMADS9* increased in large green stage and *VmSHP* in ripening fruit, indicating their differential roles in bilberry fruit. The *PyMADS18* has been suggested to be involved in the regulation of anthocyanin biosynthesis in pear (Wu et al., 2013). During the fruit ripening, expression of *PyMADS18* was shown to increase at early stages of development, after that decreasing until it was up-regulated again at the end of fruit maturation period (Wu et al., 2013) resembling the expression pattern obtained in this study for *VmMADS18*.

*SEPALLATA* (*SEP*)-like MADS-box TFs have been indicated to play central roles in ripening of both climacteric and non-climacteric fruits, best known example being *LeMADS-RIN* (Seymour et al., 2011). *FaMADS9*, a fruit-related *SEP1/2-like* gene was indicated as a positive regulator of both development and ripening of strawberry fruit with its expression up-regulated at white stage of strawberry fruit development (Seymour et al., 2011). Silencing of the gene led to inhibition of normal development of strawberry fruit (Seymour et al., 2011). The gene was shown to be ABA-inducible later by Daminato et al. (2013). Also *FaSHP*, a C-type MADS-box gene belonging to a SHATTERPROOF group was indicated as a positive regulator of strawberry fruit ripening with its expression induced by ABA (Daminato et al., 2013; Medina-Puche et al., 2016). The expression of the *FaSHP* increases in strawberry fruit after large green stage due to ABA control being highest at pink stage (Daminato et al., 2013) similarly to *VmSHP* shown in our study. Overall, our results indicate that *VmMADS18, VmMADS9*, and *VmSHP* have potential roles in the ABA-regulated bilberry fruit development and ripening.

The fourth studied MADS-box gene in the present study was *VmTDR4* belonging to a SQUAMOSA group. *VmTDR4* was earlier demonstrated to have a role in the anthocyanin accumulation during bilberry fruit ripening with its expression especially associated in flesh of ripening fruit (Jaakola et al., 2010). Silencing of *VmTDR4* in bilberry fruit resulted in chimeric berries with decreased expression of *CHS, DFR*, and *ANS* but elevated expression of *ANR* indicating modulation of flavonoid pathway through flavonoid regulating MYB TFs (Jaakola et al., 2010). Also tomato homolog for *TDR4* induced anthocyanin biosynthesis when expressed in *Arabidopsis* siliques (Jaakola et al., 2010). The response of *VmTDR4* to ABA has not been studied earlier but the present study demonstrates that *VmTDR4* is highly induced by ABA indicating its role in the ABA-regulated fruit ripening associated anthocyanin accumulation.

Also NAC family TFs have been proposed as activators of fruit ripening with some of their expression shown to be increased by ABA (Zhu et al., 2014; Jiang et al., 2017; Moyano et al., 2018). Recently, a *PpBL* gene (BLOOD) was indicated as a key regulator of anthocyanin biosynthesis in maturing blood-fleshed peach fruit (Zhou et al., 2015). It was shown to act as a heterodimer with another NAC family member, *PpNAC1* (Zhou et al., 2015). In our study, the closest bilberry homolog for the peach *BL* gene, *VmBL*, showed a significant induction in its expression by ABA treatments as well as increased expression at the onset of bilberry fruit ripening slightly before *VmTDR4*. This suggests that *VmBL* could have a role in the regulation of bilberry fruit ripening and/or anthocyanin biosynthesis through ABA-mediated signaling.

## Conclusion

This is the first report regarding the role of ABA and sugars on the regulation of bilberry fruit ripening. By using both pre- and post-harvest experiments and a molecular approach, we showed that ABA is an important positive regulator of bilberry fruit ripening processes, inducing anthocyanin biosynthesis and fruit softening. However, based on our results, sugars (glucose, fructose, and sucrose) have minor roles in the regulation of bilberry fruit ripening as sugars failed to induce anthocyanin or ABA biosynthesis in bilberry fruit but could elevate expression of some genes associated with cell wall modification. Moreover, sucrose did not enhance the effect of ABA in ripening responses. Our results suggest that the ripening regulation may be different in bilberry fruit compared to the current model of non-climacteric fruit ripening, strawberry, in which the coordinated regulation by the two signaling molecules, ABA and sucrose, have been proposed to have a key role in fruit ripening.

## Author Contributions

KK and LJ designed the experiments. KK was responsible for conducting the experiments and analyses. PT and KK conducted pre-harvest ABA treatments and subsequent gene expression analyses. KK was responsible for the writing of the manuscript with contribution of LJ and HH. All authors read and approved the final manuscript.

## Conflict of Interest Statement

The authors declare that the research was conducted in the absence of any commercial or financial relationships that could be construed as a potential conflict of interest.
