# Supplementary material for: Abscisic Acid Regulates Anthocyanin Biosynthesis and Gene Expression Associated With Cell Wall Modification in Ripening Bilberry (Vaccinium myrtillus L.) Fruits
Source: Front Plant Sci. 2018 Aug 29;9:1259. doi: 10.3389/fpls.2018.01259 (PMC6124387; doi:10.3389/fpls.2018.01259)
Supplement: Supplementary file 1 [file Image_1.pdf]

Supplementary Material

Abscisic acid regulates anthocyanin biosynthesis and gene expression associated with cell wall modification in ripening bilberry (*Vaccinium myrtillus* L.) fruits

Katja Karppinen[1,2], Pinja Tegelberg[1], Hely Häggman[1] and Laura Jaakola[2,3*]

[1] Department of Ecology and Genetics, University of Oulu, Oulu, Finland
[2] Climate laboratory Holt, Department of Arctic and Marine Biology, UiT the Arctic University of Norway, Tromsø, Norway
[3] Norwegian Institute of Bioeconomy Research (NIBIO), Ås, Norway

*** Correspondence:** Laura Jaakola, laura.jaakola@uit.no

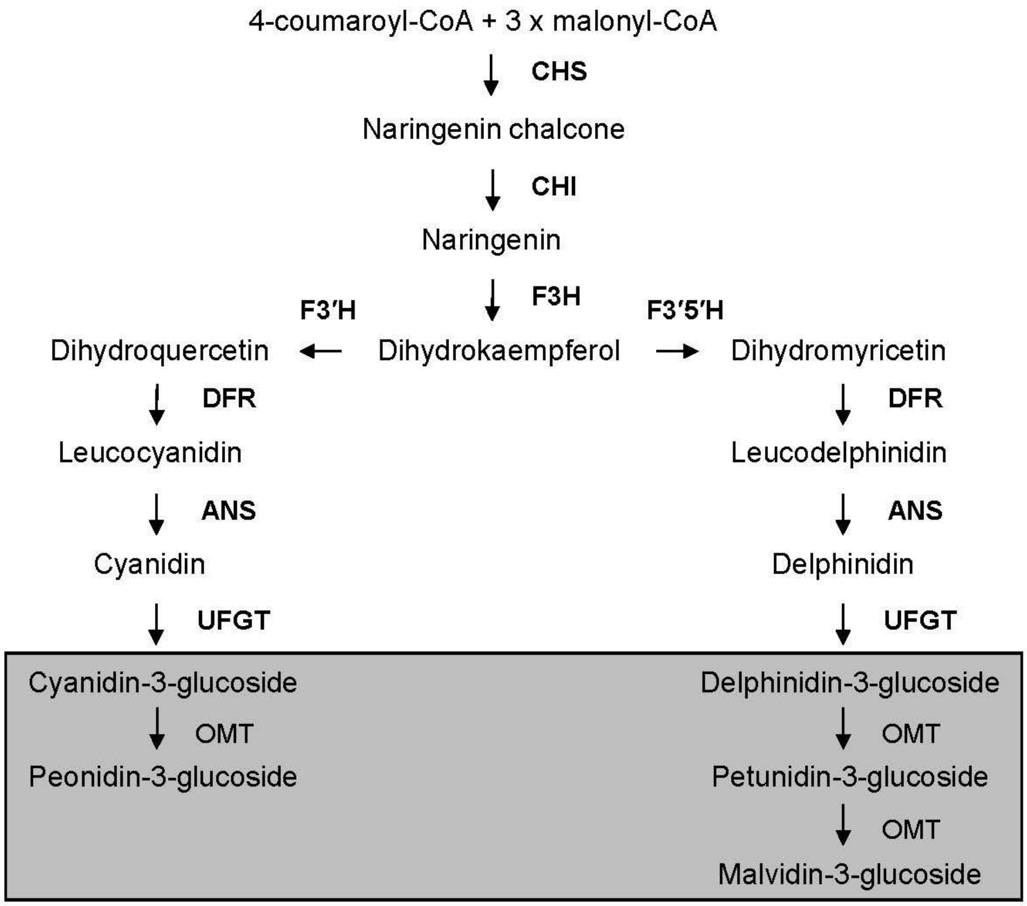

Supplementary Figure S1. Anthocyanin biosynthetic pathway. CHS, chalcone synthase; CHI, chalcone isomerase; F3H, flavanone 3-hydroxylase; F3′H, flavonoid 3′-hydroxylase; F3′5′H, flavonoid 3′5′ hydroxylase; DFR, dihydroflavonol 4-reductase; ANS, anthocyanidin synthase; UFGT, UDP-glucose: flavonoid 3-*O*-glucosyltransferase; OMT, *O*-methyl transferase.

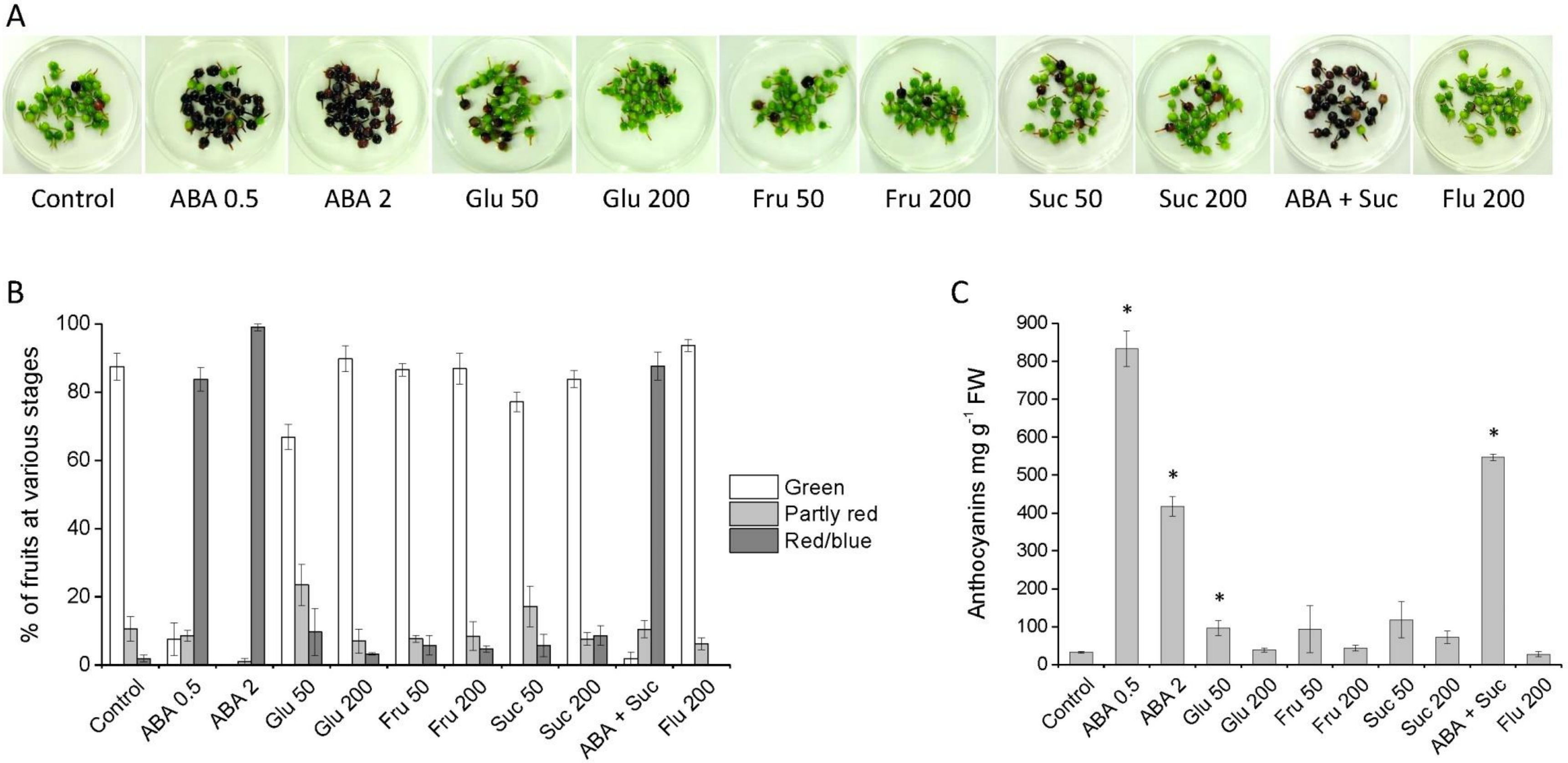

Supplementary Figure S2. Effect of six days post-harvest ABA and sugar treatments on bilberry fruit color and anthocyanin accumulation. Detached unripe green berries were immersed into solutions containing ABA (0.5 mM, 2 mM), glucose (50 mM, 200 mM), fructose (50 mM, 200 mM), sucrose (50 mM, 200 mM), 0.5 mM ABA + 200 mM sucrose, 200 µM fluridone or water (control). After 6 days from the beginning of the experiment, fruits in Petri plates **(A)** were evaluated for their color **(B)** and measured for their anthocyanin content **(C)**. Total anthocyanin content is expressed as milligrams of cyanidin-3-glucoside equivalents g^{-1} FW. Values represent means ± SEs of three replicates. Asterisks indicate significant differences from control in Student's *t*-Test ($P \leq 0.05$).

Supplementary Table S1. Gene-specific primers used for qRT-PCR analyses.

Gene	Forward primer sequence 5'-3'	Reverse primer sequence 5'-3'
VmCHS	CATGATGTACCAACAGGGTTGC	GTGATCTCAGAGCAAACCACCA
VmCHI	GTCTTTCCTCCGTCGATCAAAC	CGATCTTGCCCTTCCACTTAAC
VmF3H	GCCTGATTTCTGAGATGACTCG	CACTATTTCCCTCCAGTCTTGC
VmF3'H	TTCTTCGACACCCGAAAGTC	TCGAACCCTTTGGAATGAAG
VmF3'5'H	GATTGCGTGGATGGACTTACA	AAATCTGGGTTCCCTTTACGC
VmDFR	GAAGTGATCAAGCCGACGAT	ATCCAAGTCGCTCCAGTTGT
VmANS	GCAACTCTTCTACGAGGGCAAA	CCTGTGGAGAATGCTCTTGCAC
VmUFGT	CATCCAAACCCTGTTCCCATCC	TCATCCCTGCCTTCAAGCTCTC
VmNCED1	GCTCAAATCCACCATGATAGCC	GACCATCCAACGGTATTTCCAC
VmSS	TCAAGGAGTTGCAGACAAAGC	CAGTGCATGAGCATGGTACAC
VmSPS1	CTCACAACTCTGGTCAAAGCA	ATGCCACTTGACCGTGTAGAT
VmSPS2	TGGAGTCAGGAGGCTTTAATC	AAGTCTGGAATGACAGGAAGC
VmSPS3	CCGTCCATTGAGAGAACTTGCT	CCACTTGCCCGTACAGATCATA
VmPE1	GAGGATTTCGCACTTGATACCC	GCCACAGTGAAGTTGTTGACCT
VmPE2	ATGAATTATGGGCCCGGCTCT	GTAACCACGACGACCCGAAAA
VmPL	GTCTACGGCAATTACCACTTCC	GTAGGCGATAGTGACTTGCATC
VmPG1	GCCAGAGCTTGGCAGTTAAAGT	GGAAGGTCTCACTGCAAGCAAA
VmPG2	AGTTGGAGGAAGGTACATCAGC	TATCTGGATGGTGTCTGAGTGC
VmRGLyase	TCAATACTGTGGTGGTTGGAG	CTAACAAAGCCACGTTGATCC
VmβGAL1	GGATTGGACCCTTCGTTTGTG	CGCCTTGAAAGGCTCATTGTC
VmβGAL2	GTTCGGAGGTCCAGTTCCTTAT	CATCGAGAGGAGCATCATAGTC
VmXTH	GAGAAGACAGATTGGTCCAAGG	TCCGGTACTGAAAGGAGTCAAG
VmCEL	ACATGGTAGGATACGGTGAACG	TAATATAGAGACCCCGCCTTGC
VmXYL	GGCGAGTGTTATGTGCTCTTAC	CACAGTCCGAAACTATGTACCC
VmEXP1	CATGCAGGAAACAGGGAGGTA	TTCTGGTACCCTTCACGCTCA
VmEXP2	ACTGGCAGAGCAATTCCTACCT	GGACTCCCTTCGAAAGTACTGA
VmEXP3	AAGGGTGCCCTGTATGAAGAAG	CCAGTTTCTAGACATGGGTTGC
VmSCL8	CATGACGTCTGCCCTCAAATC	AACACGGCGATGTCTCGTACA
VmMADS18	CAGCAACCATAGCAACATGCTC	GAGATCGCTGGAGGGTTCATAA
VmMADS9	GTTCTCTGTGATGCTGAGGTTG	TCCAGTGATCCATAGCTACACC
VmSHP	ACTCCTCCCATACTGGGTCAAT	TCCCTGAGAGACATAGAGCACA
VmBL	AGACGGAGGCGATCATCATAA	ATAGCGGGATCTGAGGGTAAA
VmTDR4	CACCTTGACCCTGAGAGAGC	GTCCACCTTGGTTTTGTTGC
VmGAPDH	ATGAAGCAGCTCTTCCACCTCT	GCTGTACCACAAACTGTCTTGC
